# Diarrhea and Acute Tubular Necrosis Mimicking Hemolytic Uremic Syndrome in a Man With Immunoglobulin A (IgA) Nephropathy

**DOI:** 10.7759/cureus.15369

**Published:** 2021-06-01

**Authors:** Jayaram Saibaba, Jayachandran Selvaraj, Stalin Viswanathan, Vivekanandan Pillai, Bheemanathi H Srinivas, Jharna Mandal

**Affiliations:** 1 Department of General Medicine, Jawaharlal Institute of Postgraduate Medical Education and Research, Pondicherry, IND; 2 Department of Pathology, Jawaharlal Institute of Postgraduate Medical Education and Research, Pondicherry, IND; 3 Department of Microbiology, Jawaharlal Institute of Postgraduate Medical Education and Research, Pondicherry, IND

**Keywords:** iga vasculitis, thrombotic microangiopathy, hemolytic uremic syndrome, thrombotic thrombocytopenic purpura, shiga toxin

## Abstract

Immunoglobulin A (IgA) nephropathy is a heterogeneous disease with variable clinical presentations ranging from asymptomatic hematuria to advanced renal failure. A young male diagnosed with IgA vasculitis (skin, joints, and gastrointestinal) one month ago and placed on oral steroids presented with acute diarrhea, hemolytic anemia, renal failure (non-dialysis requiring), altered sensorium, and thrombocytopenia. The stool was found to be positive for Shiga toxin. He improved with methylprednisolone pulse alone, and renal biopsy showed acute tubular injury.

## Introduction

Thrombotic microangiopathy (TMA) is a pathological syndrome with the clinical presentation of nonimmune (Coombs-negative) microangiopathic hemolytic anemia (MAHA), thrombocytopenia, and organ dysfunction [1]. Acute kidney injury is common in TMA, while extrarenal manifestations involve the brain, lungs, pancreas, and liver [2]. Hemolytic uremic syndrome (HUS) is a severe form of TMA that could be hereditary or acquired [2]. TMAs can be either primary (e.g., thrombotic thrombocytopenic purpura [TTP]) secondary, or infection-associated (e.g., HUS) [1]. Immunoglobulin A (IgA) vasculitis/Henoch-Schönlein purpura is a disorder that causes inflammation and bleeding of the small blood vessels of the skin, joints, intestines, and kidneys [3]. TTP is a blood disorder in which small blood clots are formed all over the body, resulting in vascular, renal, and cerebral dysfunction [4]. Herein, we report a patient with Shiga toxin-positive acute diarrhea, acute kidney injury, and altered sensorium, mimicking TMA, but the renal biopsy was suggestive of acute tubular necrosis (ATN).

## Case presentation

**Figure 1:**
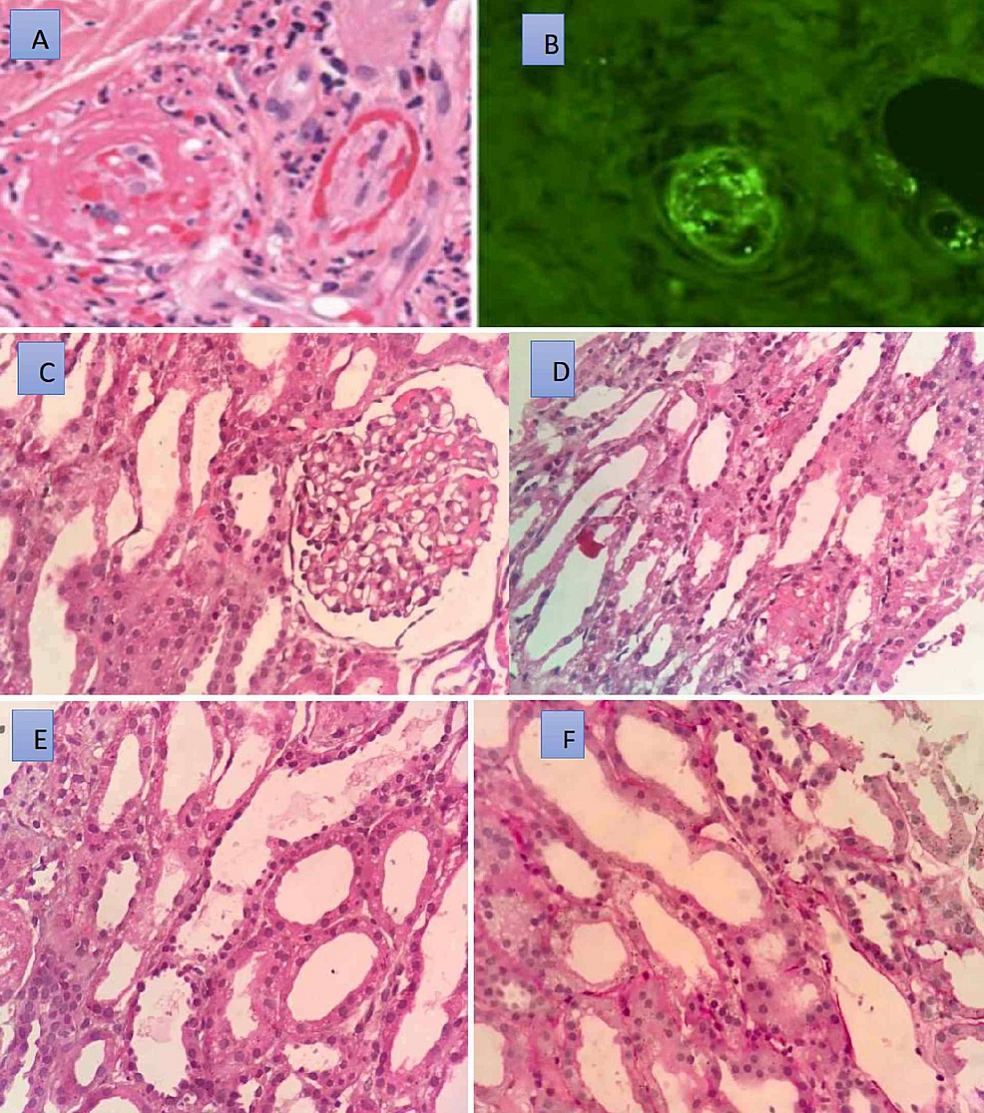
Histopathology of the skin and the kidney (A) Dermal small vessel vasculitis showing fibrin, neutrophils, and karyorrhectic debris within and surrounding vessel walls with extravasated erythrocytes (H&E; 400x). (B) Granular deposition of IgA within vessel walls. (C) Glomeruli is histologically within normal limits (H&E; 400x). (D)  The lumen of the tubules shows shedded tubular epithelial cells along with RBCs. Adjacent tubules show mild ATN changes (H&E; 400x). (E) Arrows show shedded tubular epithelial cells (H&E; 400x). (F) PAS stain highlighting the damaged tubular epithelium (400x). H&E - hematoxylin and eosin; IgA - immunoglobulin A; RBC - red blood cells; ATN - acute tubular necrosis; PAS - periodic acid–Schiff

A 21-year-old male working in a metal store in Chidambaram, South India, was diagnosed one month prior to his current admission with IgA vasculitis after he had palpable purpura (Figures 1a, 1b), polyarthritis, and colicky abdominal pain for three weeks. Serology and viral markers were negative. Upper gastrointestinal endoscopy showed severe gastropathy with erosions. Urinalysis revealed protein 1+ and 20-25 red blood cells (RBCs) but no dysmorphic RBCs. Serum creatinine was normal. He was initiated on oral prednisolone 60 mg on the third day of admission and discharged on the seventh day with a plan to taper the prescribed steroids on an outpatient basis.

Two weeks after discharge, the patient had lunch consisting of rice, lentil pancakes, and chicken biryani at a wedding feast. Three hours later, he developed the following symptoms: watery stools without blood or mucus (~10 episodes/d), non-bilious vomiting (3-4 episodes/d), periumbilical abdominal pain for one day, and slurred speech on the night before he was admitted to our hospital. There was no fever or other gastrointestinal, respiratory, or neurological complaints. The patient was a teetotaller and a nonsmoker. The other wedding guests showed no gastrointestinal symptoms.

On examination in the emergency department, he was afebrile and had a pulse rate of 130 beats per min, unrecordable blood pressure, a respiratory rate of 22 breaths/min, pedal edema, pallor, dry mouth and tongue, sunken eyes, and disorientation to place and time, with no focal neurological deficits. The lungs were clear on auscultation. The laboratory values are given in Table 1.

Hypovolemic shock with acute kidney injury, TMA due to either HUS or TTP, and IgA vasculitis with neurological dysfunction were considered the differentials. Opening central venous pressure was 3 cm, and fluids were administered according to the central venous pressure. Ceftriaxone 2 g intravenously OD was initiated for diarrhea. Loose stools and vomiting frequency reduced from the second day onward. Stool examination for ova and cysts was negative. Following fluid resuscitation, the blood pressure improved. However, the creatinine trend worsened, and the altered sensorium deteriorated, leading to visual hallucinations and agitation. The ultrasonogram of the kidneys was normal. Lumbar puncture was initially deferred, considering that the altered sensorium was probably due to hypovolemic shock and renal dysfunction. His urine output 48 h after admission was 900 mL. Then the patient was initiated on pulse methylprednisolone on the third day (1 g x three days) for probable IgA disease flare that could cause neurological and renal dysfunction.

**Table 1:** List of investigations during hospital stay and follow-up LDH - lactate dehydrogenase; PCR - protein creatinine ratio; ANA - antinuclear antibody; ANCA - antineutrophil cytoplasmic antibodies; CSF -cerebrospinal fluid; scrub typhus PCR - polymerase chain reaction; HSV - herpes simplex virus; JE - Japanese encephalitis

Date	Day1	Day2	Day3	Day4	Day5	Day6	Day7	Day8	Day9	Day10	Day11	Follow up
Urea (mg/dL)	99	144	158	135	162	168	132	115	105	92	83	28
Creatinine (mg/dL)	5.52	6.01	5.62	4.57	3.22	2.91	2.36	1.96	2.01	1.61	1.33	0.80
Total bilirubin (mg/dL)	0.70	0.44	0.59	0.67	0.54	0.52	0.45	0.45	0.49	0.40	0.52	0.65
Direct bilirubin (mg/dL)	0.23	0.14	0.18	0.17	0.13	0.20	0.13	0.08	0.08	0.09	0.13	0.15
LDH (IU/L)	563	1,062	943	993	808	663	575	481	330	331	315	
Urine PCR (<0.2)							1.49					
24 hr urine protein (mg/day)							2300					
Hemoglobin (g/L)	119	106	109	112	114	105	106	112	106	109	117	118
Platelets (x10^9^/μL)	137	92	120	144	214	211	241	201	189	215	216	292
Urinalysis	Field packed RBCs	----			20-25 RBC	8-10 RBC	-------	------	-----	----	-----	
Urine output (mL)	900	1,000			1,500	1,400	1,400	1,100	1,200	1,500	1,500	
ANA	Negative											
c-ANCA	Negative											
C-reactive protein (mg/L)	74											
Erythrocyte sedimentation rate (mm/h)	44											
C3 g/L	0.91											
C4 g/L	0.18											
Blood cultures	Sterile						Sterile					
Urine cultures	Sterile						Sterile					
CSF fluid cell count (lymphocyte percentage)					10 (100)							
CSF glucose mg/dL					126							
CSF protein g/dL					56							
CSF culture					Sterile							
Encephalitis panel (scrub typhus PCR, HSV PCR, JE PCR)				Negative								
CSF GeneXpert				Negative								

Plasma exchange (PLEX) was also planned because of Coombs-negative hemolytic anemia, thrombocytopenia, altered sensorium, renal dysfunction, and a PLASMIC score of 5%. Multiple peripheral smears could not find schistocytes, and hence TTP was considered less likely. Activated partial thromboplastin time, prothrombin time, and fibrinogen were normal. Creatinine, platelets, and lactate dehydrogenase (LDH) began to improve on the fourth to fifth days while diarrhea stopped on the fourth day. Workup for scrub typhus, malaria, HIV, dengue, and bacterial endocarditis was negative. Though hallucinations and agitation subsided, the patient remained confused. Since the laboratory reports were improving as was the sensorium, PLEX was deferred; lumbar puncture and MRI were performed on the fifth day. Stool returned positive for Shiga toxin on the fifth day. The next day, the sensorium spontaneously recovered.

The patient was switched back to oral prednisolone 60 mg after three days of methylprednisolone. Renal biopsy, which had been deferred because of thrombocytopenia and altered sensorium, was performed on the 10th day and was reported to be a diffuse tubular injury (Figures 1c-1f). He was discharged on the 12th day of admission with advice to continue oral prednisolone. He returned a month later to the outpatient department, and his creatinine was 0.80 mg/dL, while urinalysis was normal. He had no further complaints.

## Discussion

We report a young male with gastrointestinal, hematological, and renal abnormalities, which developed after he attended a feast. The patient had been previously diagnosed with IgA vasculitis. Acute kidney injury was the most severe manifestation, followed by diarrhea, altered sensorium, thrombocytopenia, and anemia. Anemia was a mild drop in hemoglobin of 130 g/L from the time of the previous admission and could also be explained by rehydration and improvement of hemoconcentration. LDH elevation made us consider MAHA, but schistocytes could not be demonstrated on multiple peripheral smears, and the bilirubin was also normal. Most TMAs usually have MAHA and thrombocytopenia, and TMA is usually evident in kidney biopsy. HUS without MAHA or other evidence of hemolysis, such as LDH or bilirubin elevation, has rarely been reported in the literature [5]. Thrombocytopenia was mild, without a bleeding diathesis or requirement of blood products. Other workups for fever and disseminated intravascular coagulation turned negative. The three initial differential diagnoses considered were hypovolemic shock with ATN, HUS/TTP, and immunoglobulin A-associated vasculitis (IgAV) with neurological dysfunction.

First, altered sensorium was attributed to shock and renal dysfunction. With the further worsening of altered sensorium and creatinine in the hospital, steroids had been initiated for probable IgAV and were found to be beneficial. Cerebrospinal fluid studies and MRI were normal. Acute kidney injury following diarrhea and shock was considered probable HUS or ATN; the Shiga toxin report (albeit late) supported our diagnosis of a probable TMA with acute kidney injury, but the renal biopsy was suggestive of acute tubular injury. The unusual findings regarding Shiga-toxin-producing *Escherichia coli*-related HUS in our patient were his older age, the absence of abdominal pain and bloody diarrhea, and the persistence of diarrhea after the onset of renal failure [2]. Other acquired infectious causes of HUS include *Streptococcus pneumoniae*, HIV, H1N1 influenza, and mycoplasma [2].

HUS/TTP was considered the second possibility. HUS with IgAN has rarely been reported, with improvement following PLEX [6]. A report from Chandigarh described a young woman with HUS and cortical necrosis due to IgA nephropathy (IgAN) [6]. A case report from France had described a middle-aged hypertensive presenting with diarrhea, followed by altered consciousness and renal failure, with elevated LDH, schistocytes, and a “fleeting thrombocytopenia,” but without anemia or hyperbilirubinemia. The patient did not improve with either eculizumab or PLEX that was administered for probable atypical HUS with TMA but surprisingly recovered with methylprednisolone. The biopsy was finally suggestive of IgAN [7]. Eculizumab was not available in our institution; PLEX was kept pending by the ICU services since MAHA could not conclusively be proven on the peripheral smear. TTP was considered (PLASMIC score 5%), except that the renal failure was more severe than neurological dysfunction and there was no fever making TTP less likely. ADAMTS13 testing was not available at our institution. PLEX was finally deferred as there was a significant improvement in renal and hematological parameters on the second day of methylprednisolone itself.

Microscopic hematuria at admission with subnephrotic range proteinuria (performed within two days of initiating methylprednisolone) and improvement of sensorium with steroid therapy favored IgAN, the third differential. We do not know whether steroids influenced the renal biopsy findings since the procedure was performed on the 10th day, and there was no evidence of TMA on the biopsy. Disorders such as antineutrophil cytoplasmic antibodies-associated vasculitis, IgA vasculitis, and membranous nephropathy may be associated with TMA [1]. The findings of TMA can be observed in over half of patients with IgAN [8,9]. Also, microbial factors contribute to secondary IgAN by triggering the generation of pathogenic IgA and the deposition of IgA in the mesangium.

## Conclusions

TMA due to HUS or TTP may coexist with IgA vasculitis. Our case is an unusual scenario-where diarrhea-related hypovolemic shock, ATN, and IgAN flare mimicked TMA, was probably associated with Shiga-toxin-producing *E. coli*-related HUS and improved with steroids alone. Renal biopsy in IgA vasculitis can help diagnose the etiology of renal failure.
